# Bibliometric analysis for artificial intelligence in the internet of medical things: mapping and performance analysis

**DOI:** 10.3389/frai.2024.1347815

**Published:** 2024-08-12

**Authors:** Haruna Chiroma, Ibrahim Abaker Targio Hashem, Mohammed Maray

**Affiliations:** ^1^College of Computer Science and Engineering, University of Hafr Al Batin, Hafar Al Batin, Saudi Arabia; ^2^College of Computing and Informatics, Department of Computer Science, University of Sharjah, Sharjah, United Arab Emirates; ^3^Department of Computer Science, Department of Information Systems, King Khalid University, Abha, Saudi Arabia

**Keywords:** artificial intelligence, artificial general intelligence, bibliometric analysis, explainable artificial intelligence, generative artificial intelligence, internet of medical things, sensors

## Abstract

The development of computer technology has revolutionized how people live and interact in society. The Internet of Things (IoT) has enabled the development of the Internet of Medical Things (IoMT) to transform healthcare delivery. Artificial intelligence has been used to improve the IoMT. Despite the significance of bibliometric analysis in a research area, to the best of the authors' knowledge, based on searches conducted in academic databases, no bibliometric analysis on artificial intelligence (AI) for the IoMT has been conducted. To address this gap, this study proposes performing a comprehensive bibliometric analysis of AI applications in the IoMT. A bibliometric analysis of top literature sources, main disciplines, countries, prolific authors, trending topics, authorship, citations, author-keywords, and co-keywords was conducted. In addition, the structural development of AI in the IoMT highlights its growing popularity. This study found that security and privacy issues are serious concerns hindering the massive adoption of the IoMT. Future research directions on the IoMT, including perspectives on artificial general intelligence, generative artificial intelligence, and explainable artificial intelligence, have been outlined and discussed.

## 1 Introduction

The technology prediction prepared by the IEEE Computer Society for the year 2023 clearly indicated that artificial intelligence (AI) is the dominant research field for the future compared to other technology research areas, with most of the AI research areas having a high likelihood of success and a significant correlation to human impact (IEEE Computer Society, 2023). AI has been dominating media and general public discussion recently because of its integration into almost every space of society. AI-based systems are now perceived as autonomous agents rather than mere tools (Dignum, 2018). As a result, AI has transformed the Internet of Things (IoT).

Although not a new concept, IoT remains a hot research topic. It is estimated that by 2030, over 29 billion devices will be connected globally through the IoT, up from over 9 billion in 2020. This represents almost a threefold increase. China is also projected to have the highest number of IoT-connected devices, approximately 5 billion (Statista, 2023). The IoT has penetrated almost all aspects of society, including, but not limited to, health, transportation, agriculture, security surveillance, and waste management, fundamentally changing how people live and interact with the environment. The integration of IoT with the medical field has given rise to the Internet of Medical Things (IoMT).

The IoMT is an emerging concept in the medical domain that is gaining significant attention from the research community. The IoMT uses information and communication technology to connect smart medical devices and applications and share data. These smart medical devices and applications monitor and continuously sense patients' health status and transfer the data to physicians for decision-making. As a result of the massive number of devices connected to the IoMT, a vast volume of high-velocity, high-veracity medical data is collected, referred to as big data (Manogaran et al., 2018). The IoMT has laid the foundation for personalizing intelligent and reliable precision medicine (Bogdan et al., 2022) and ultrasound networks (Santagati et al., 2020).

For these data to be valuable and provide insight, they need to be processed using advanced AI algorithms to improve medical practice, enhance IoMT functionalities, and develop intelligent medical systems. A prominent AI technique used in IoMT is machine learning, especially deep learning (DL), due to its ability to handle large volumes of data. Unlike shallow intelligent algorithms, whose performance degrades with increasing amounts of data, DL thrives with more data. The possible reason machine learning takes center stage in the IoMT is likely because of the massive amount of data associated with the IoMT. The core of machine learning is data—without data, there is no machine learning. It is believed that data could be the crude oil of the future. Many researchers have applied DL and other AI techniques to solve various problems in the IoMT, leveraging the extensive data generated by these systems.

For example, Zhang et al. (2020) introduced DL to create an IoMT framework for elderly healthcare. Jiang et al. (2022) used deep reinforcement learning for energy savings in the IoMT. Xuan and You (2020) applied a convolutional neural network in IoMT to diagnose pancreatic tumors. Khan and Akhunzada (2021) used hybrid DL to detect malware in IoMT. Ghosal et al. (2022) proposed a hybrid algorithm for detecting anemia disease in IoMT. Many studies applying machine learning to IoMT exist in the literature, though providing details for all of them is beyond our scope. Other aspects of AI penetrating IoMT include data mining (Toor et al., 2020), robotics (Gatouillat et al., 2018), and natural language processing (Lin and Wu, 2021).

It is imperative to conduct a bibliometric analysis to analyze the current trends of AI applications in the IoMT literature. Such an analysis can provide guidance and motivation for conducting future research, helping to develop the research area further. Bibliometric analysis can outline the overall structure and general applications of AI in IoMT. Several researchers have published bibliometric analyses to show research trends in specific fields. Chiroma et al. (2020) conducted a bibliometric analysis of machine learning applications in diagnosing COVID-19, focusing specifically on machine learning. Muhuri et al. (2019) carried out a bibliometric analysis for Industry 4.0. Ezugwu et al. (2021a) performed a classification and bibliometric analysis of nature-inspired algorithms for solving global optimization problems. Shukla et al. (2019) conducted a bibliometric analysis of AI applications in engineering.

Despite the high volume of publications on AI in IoMT, to the best of the authors' knowledge, no bibliometric analysis has been conducted to outline the overall structure of AI applications in IoMT.

In this article, we propose to conduct a comprehensive bibliometric analysis, performance analysis, and scientific mapping of AI applications in the IoMT to show current trends and future expectations, thereby motivating future development in this field.

The summary of the contributions of the study is outlined as follows:

- A thorough bibliometric analysis within the scope of AI in IoMT has been performed.
- The study found trending topics, prolific authors, top influential authors, the impact of the research area, countries and publication distribution, top disciplines, author keywords, and top sources of AI for IoMT literature.
- The study discusses highly productive author institutions, author countries, discipline areas, corresponding author countries, and collaborations, and it has shown the top performers in each category.
- The research growth of AI in IoMT over a period of 7 years was analyzed and documented.
- The visualization of the mapping network structure for co-authorship, collaborating countries, co-keywords, and commonly used keywords by the authors working in this field is shown, as extracted from the academic database indexing the literature.
- The study unraveled the past, present, and future of AI in IoMT based on a large-scale bibliometric analysis corpus.

Other sections of the article are organized as follows: Section 2 presents literature reviews, pointing out the motivation for the current article. Section 3 provides details on the bibliometric methodology. Section 4 discusses the findings of the study, which includes both scientific mapping and performance analysis. Section 5 points out limitations and suggestions for future works before concluding in Section 6.

## 2 Previously published studies and motivation for the current study

This section discusses already-published studies in the area of the IoMT and the motivation for the current study. Dwivedi et al. (2022) published a review highlighting the role of the IoMT in improving the medical system, including its benefits to patients. The review outlined and discussed the technologies providing support to the IoMT. It was found that the IoMT mitigates barriers hindering the smooth operation of healthcare delivery, including, but not limited to, telemedicine, remote monitoring, sensors, and robotics. However, the article revealed significant challenges to the widespread adoption of the IoMT, including privacy and security issues, handling of large datasets, scalability, cost efficiency, power consumption, data management, interoperability, and regulatory framework. Ashfaq et al. (2022) presented a review of the studies conducted by the research community in developing the IoMT. They discussed sensors and communication technologies used in the IoMT to deliver healthcare services. The integration of machine learning frameworks into the IoMT was covered in areas such as disease prediction, remote health monitoring, human behavior prediction, automatic insulin injection, intelligent medicine boxes, mental health monitoring, sleeping monitoring, seizure detection, fall detection, and stress and anxiety monitoring. The IoMT is known for its extensive data collection through the sensors embedded in the systems. The review found major challenges in the IoMI, specifically data security and prediction accuracy.

Kagita et al. (2022) conducted a rigorous review of the efforts made by researchers to address privacy and security issues in the IoMT. They identified the main challenges hindering the full success of the IoMT as related to security and privacy. Rasool et al. (2022) provided a comprehensive review of these issues, creating a taxonomy for the types of IoMT devices vulnerable to attacks and the protocols for defending these devices. The study proposed requirements for the development of security solutions in the IoMT, revealing that sustainable security solutions are the main challenge in the IoMT. Al-Turjman et al. (2020) presented a review detailing the status of IoMT development. The study reviewed body- and object-centric applications and covered data mining applications, mainly both supervised and unsupervised methods in the IoMT. The challenges identified in their review included cost, security and privacy, precision and accuracy, safety, energy consumption, and usability. In summary, there are several technical and design challenges associated with the IoMT. Bigini et al. (2020) presented a survey on user-centric approaches combining blockchain and IoMT to give users absolute control over their data and ownership. However, the study was limited to those promoting the control of user data by the surveyed users and found that creating a fully decentralized user-centric system for data control in the IoMT remains a challenge. Gatouillat et al. (2018) provided a rigorous literature review on the contributions of cyber-physical systems to the IoMT. They discussed the applications of democratized medical devices from the perspective of healthcare service providers and patients. Reliability, medical device validation, accuracy, and service-oriented IoMT were identified as challenges in the IoMT. Hameed et al. (2021) discussed the use of machine learning approaches in providing security and privacy solutions in the IoMT while maintaining the quality, services, and lifespan of medical devices. They found that a significant number of machine-learning approaches were deployed in the IoMT to protect the network layer and medical devices. However, researchers mainly focus on enhancing conventional metrics without considering the complexity of the metrics for evaluation. The data mostly used in the research barely represent the IoMT data and environment. Resource complexity and use of power can limit the effectiveness of machine-learning approaches if ignored.

Sun et al. (2019) surveyed the security and privacy requirements for the IoMT. The security and privacy issues surveyed were cut across data levels, sensor levels, personal servers, and medical servers. The main challenges identified were malicious cyberattacks on the IoMT ecosystem, and most studies focus on simulation solutions rather than real-world practical work. Bajaj et al. (2021) provided an overview of the recent developments in the IoMT. Security and privacy were identified as major obstacles to the IoMT. Hasan et al. (2022) presented a comprehensive survey of the measures to counter security threats and vulnerability in the 5 G-enabled IoMT. In addition, the survey covered connected clinical systems, computing systems, and sensors for medical purposes. The threats that could hamper the success, security, and privacy of the IoMT were identified. It was found that the 5 G-enabled IoMT is vulnerable to denial of service, eavesdropping, malware attacks, security, privacy, and confidentiality issues. However, cryptographic techniques can be used to improve the security aspect of the IoMT.

Malamas et al. (2021) presented a systematic survey on the risk assessment and methodologies for mitigating security risks in the IoMT. They surveyed research on IoMT risk assessment and extracted and listed the most significant mitigation controls for IoMT security. Some of the major challenges highlighted in the study include a lack of information security coverage by manufacturers, limited budgets by health organizations, data integrity issues, and abuse of human rights. Manickam et al. (2022) surveyed the applications of AI in improving the effectiveness of IoMT and medical devices, including cardiac diagnoses, cancer diagnoses, and diabetes management. In addition, the survey discussed how robotics leverage surgery for biomedical purposes. The improvements in functionality, accuracy, IoMT decision-making capacity, and risk assessment evaluation were critically examined in the survey. Major challenges identified include handling large-scale data, heterogeneity in data collection, possible connectivity failures, and noisy data.

Razdan and Sharma (2022) provided an overview of the technologies underpinning the IoMT, including its architecture. They identified blockchain, AI, unclonable functions, and software networks as technologies with the potential to address security, privacy, accuracy, and performance issues in the IoMT. The study also covered three different case studies demonstrating improvements in the IoMT based on these technologies. Sadhu et al. (2022) presented a review of the security requirements and solutions in the IoMT, focusing on cryptography, physical unclonable functions, blockchain, and named data networking. They provided an overview of the IoMT ecosystem, standards, regulations, and security mechanisms, identifying major challenges such as memory size, scalability, computational resources, overhead communication, energy consumption, and security. In another study, Kakhi et al. (2022) published a review focusing on the role of AI in developing the IoMT, discussing both software and hardware AI applications, including wearable medical devices. Interestingly, the market share of wearable devices was analyzed, with security and privacy identified as significant challenges. Joyia et al. (2017) presented an overview of the benefits, applications, and challenges facing the IoMT in healthcare delivery. The main challenges outlined in the study include privacy, security, a high volume of data, and variability. The summary of the studies is presented in Table 1.

**Table 1:** Attributes of the previously published studies on AI applications in the IoMT.

**References**	**Main focus**	**AI**	**Bibliometric analysis**		**Main challenges**
			**Performance analysis**	**Science mapping**	
Dwivedi et al. (2022)	Role of IoMT in enhancing health systems	No	No	No	Security and privacy
Ashfaq et al. (2022)	Applications of ML in the IoMT	Partial	No	No	Security and accuracy in prediction
Kagita et al. (2022)	Privacy and security	No	No	No	Privacy and security
Rasool et al. (2022)	Security and privacy	No	No	No	Sustainability of security and privacy
Hasan et al. (2022)	Security, privacy, and countermeasures	No	No	No	Security, privacy, and confidentiality
Manickam et al. (2022)	AI in IoMT	Yes	No	No	Large-scale data, heterogeneity, loss of connectivity, and noisy data
Razdan and Sharma (2022)	Security and privacy	Partial	No	No	Security, privacy, and accuracy
Sadhu et al. (2022)	Security	No	No	No	Scalability, resources, memory, communication, energy, and security
Bajaj et al. (2021)	Application and use cases	No	No	No	Security and privacy
Sun et al. (2019)	Privacy and security	No	No	No	Malicious cyberattacks and limited real-world studies
Al-Turjman et al. (2020)	Body and object-centric applications	Partial	No	No	Technical and design challenges
Bigini et al. (2020)	Blockchain in IoMT	No	No	No	Lack of fully decentralized, user-centric
Gatouillat et al. (2018)	Cyber-physical systems in IoMT	No	No	No	Issues of reliability, validation, accuracy, and service-oriented IoMT.
Hameed et al. (2021)	Machine learning for privacy and security in IoMT	Partial	No	No	Resource complexity, power usage, and device degradation.
Malamas et al. (2021)	Security assessment methodologies	No	No	No	Lack of information security coverage by manufacturers, limited budgets by health organizations, data integrity, and human rights abuse
Kakhi et al. (2022)	Applications of AI in IoMT	Yes	No	No	Security and privacy

As discussed previously, recent surveys on the IoMT from different perspectives have been published. These studies primarily focus on surveys without covering bibliometric analysis, performance analysis, or scientific mapping, as evident in the Table 1 summary. However, a literature review should be complemented by a bibliometric analysis, as argued by Donthu et al. (2021). Therefore, our study intends to address this gap by conducting a bibliometric analysis.

## 3 Methodology

**Figure 1:**
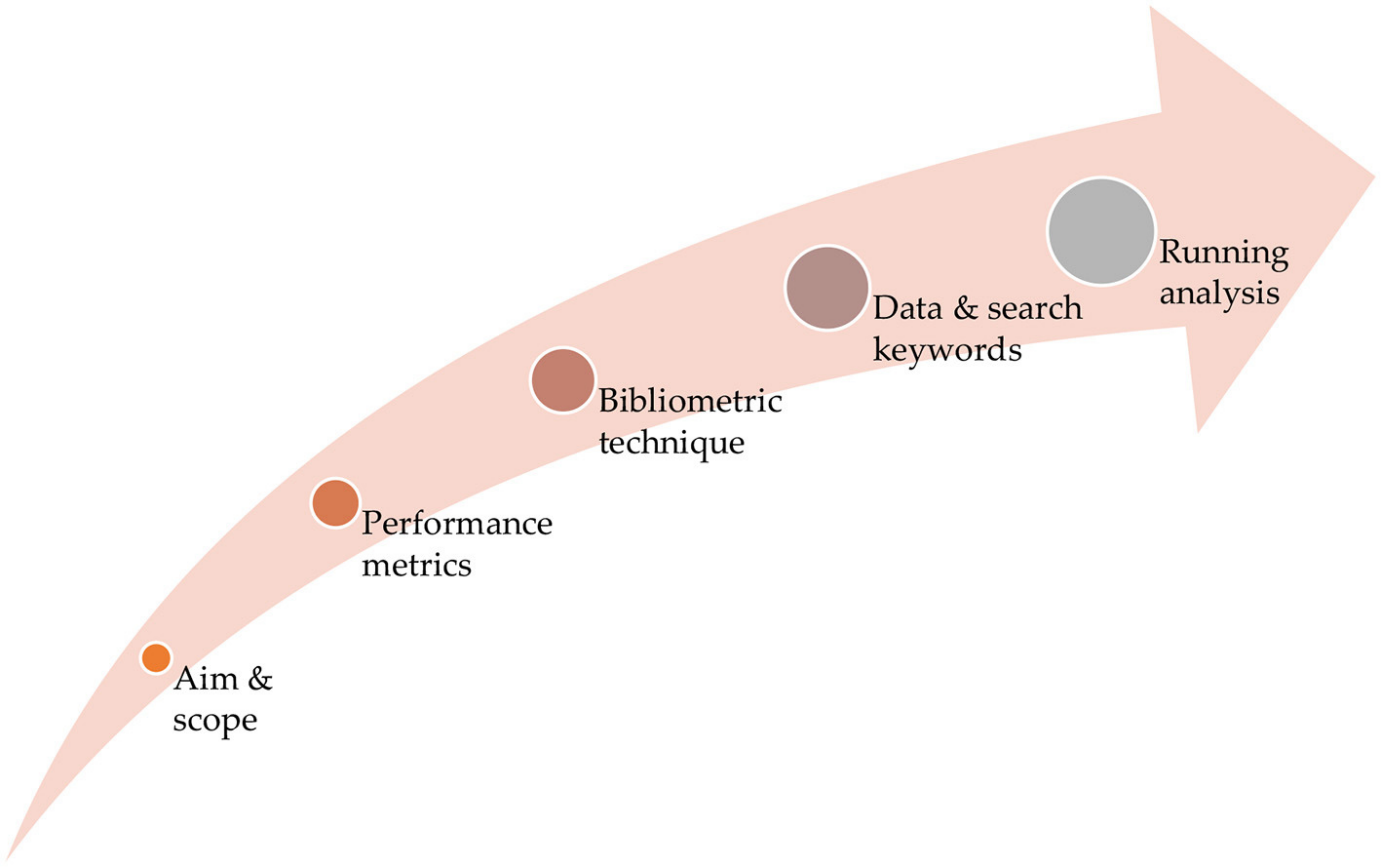
The stages in the procedure of the bibliometric analysis methodology.

The research methodology for the bibliometric analysis involves both performance analysis and science mapping of AI in the IoMT, as defined by the research objective. The bibliometric research methodology used in this study mainly follows the proposal put forward by Donthu et al. (2021). While many methodologies can be found in the literature (e.g., Weidt and Silva, 2016; Palmatier et al., 2018; Muhuri et al., 2019; Shukla et al., 2019; Ezugwu et al., 2021a), the study adopted the bibliometric guidelines proposal of Donthu et al. (2021) because they comprehensively cover every aspect of bibliometric analysis, with logically arranged and updated information compared to other bibliometric methodologies in the literature. The details of the methodology documented by Donthu et al. (2021) can facilitate the reproducibility of outcomes and the replication of the study in other research fields. The main stages of the bibliometric analysis conducted in this study are presented in Figure 1.

### 3.1 Aim and scope of the bibliometric analysis

The bibliometric analysis aims to uncover the prolific research components, such as countries, authors, journals, and institutional affiliation, from the perspective of performance analysis. From the perspective of science mapping, the study aims to make known the bibliometric structure coupling networks in the research constituent of AI for the IoMT, as well as the pertinent theme in the clusters.

The scope of the study covered the IoMT applications of AI. The IoMT and AI were selected because AI has recently generated keen interest from the media and public, and it is a hot research area. Likewise, the IoMT is an emerging research area trending in the research community. Both AI and IoMT are broad research fields suitable for conducting bibliometric analysis. There was a sufficiently large number of articles to warrant the bibliometric analysis in the research area because the number of articles returned was more than 1,000, as presented in Table 2, exceeding the benchmark of 500 set in the literature (e.g., Donthu et al., 2021). Therefore, the AI for IoMT was selected for the bibliometric analysis.

**Table 2:** Basic information about the articles published in the field of AI for IoMT.

**Description**	**Results**
**Main information about the data**	
Timespan	2016:2023
Sources (journals, books, etc.)	556
Documents	1,347
Average years from publication	2.1
Average citations per document	10.23
Average citations per year per document	2.735
**Document types**	
Article	779
Book	4
book chapter	106
conference paper	352
conference review	23
Editorial	10
Erratum	5
Note	2
Review	65
short survey	1
**Document contents**	
Keywords Plus (ID)	6,769
Author's Keywords (DE)	3,455
AUTHORS	
Authors	3,963
Author Appearances	5,772
Authors of single-authored documents	53
Authors of multi-authored documents	3,910
**Authors collaboration**	
Single-authored documents	79
Documents per author	0.34
Authors per document	2.94
Co-Authors per documents	4.29
Collaboration Index	3.08

### 3.2 Performance metrics

**Figure 2:**
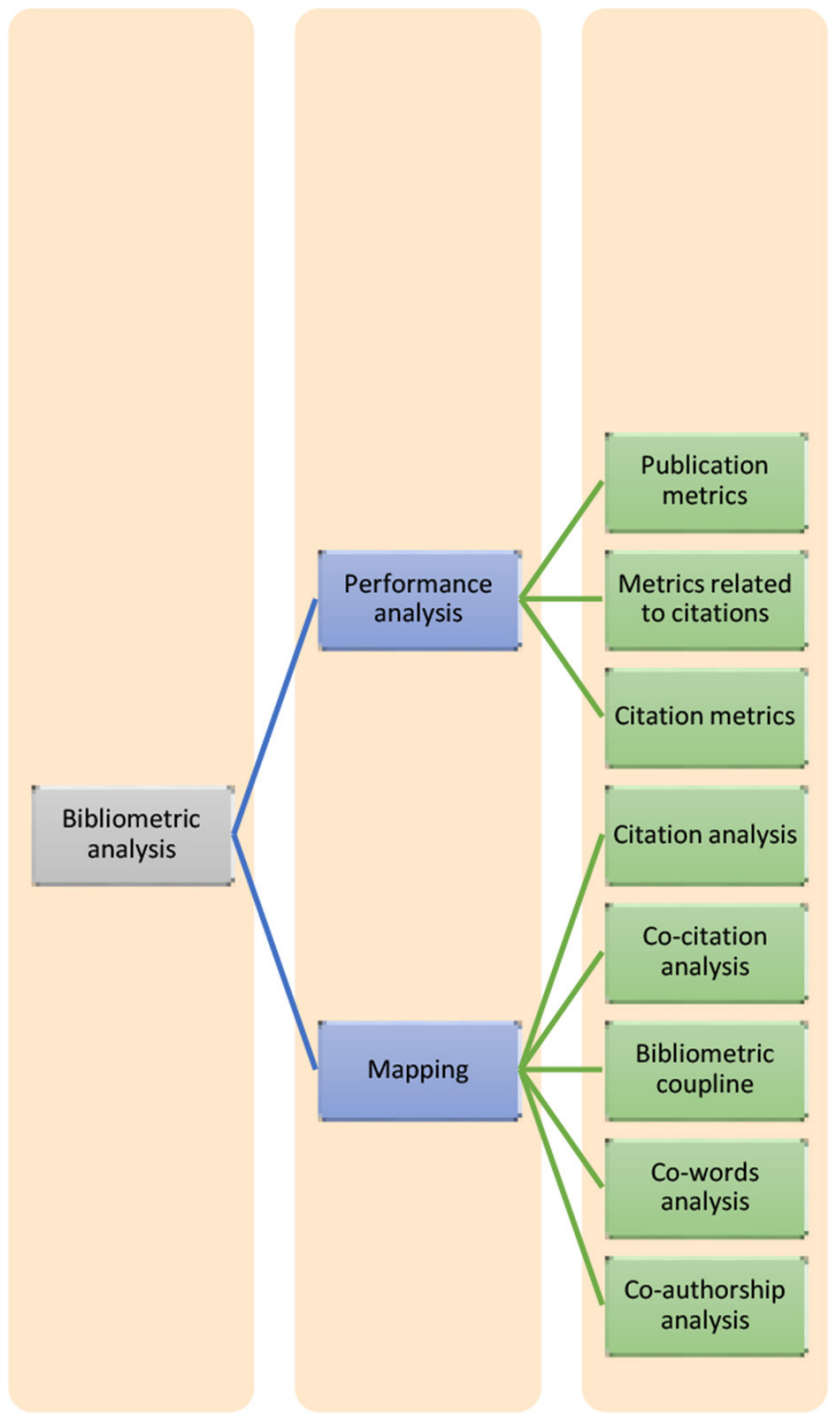
Bibliometric analysis metrics.

The prominent performance measures in the bibliometric analysis are productivity (i.e., number of publications), impact (cumulative citations), and influence of the research components per year and citation per publication. The bibliometric analysis is descriptive, but it recognizes the significance of various constituents of the research area. The performance metrics used for the mapping were as follows: citation analysis, bibliographical coupling, co-word analysis, and co-authorship analysis. The metrics for the performance analysis were in three categories: publication metrics, metrics related to citation, and citation and publication metrics (Donthu et al., 2021). Figure 2 depicts performance metrics.

### 3.3 Bibliometric analysis technique

The bibliometric analysis in this study used both performance analysis and science mapping. Therefore, the techniques selected for the study based on its aim and scope included co-citation analysis, bibliometric analysis coupling, and co-wording analysis. These techniques were used to explore the past, current state, and future of AI in the IoMT using a large bibliometric analysis corpus. To reveal general themes, co-words and author keywords were selected for analysis. For performance analysis, the profiles of participants in AI in the IoMT research field were examined, focusing on productivity and impact. This comprehensive approach aims to provide a detailed understanding of the research landscape and guide future developments in the field.

### 3.4 Search Keywords and Data Collection

This stage involved gathering data for the bibliometric analysis based on the technique chosen in the previous stage (Section 3.3). The search for the data in the database requires using specific keywords to query the database, ensuring the retrieval of a large-scale relevant dataset focused on AI in the IoMT, enough for bibliometric analysis. The keywords were coined based on the scope and aim of the study, focusing on AI in IoMT, which has the potential to return enough data. Preliminary keywords were formulated by consulting the relevant literature on AI in the IoMT and refined by brainstorming among the researchers to arrive at the final set of keywords. The final keywords used were as follows: (TITLE-ABS-KEY (“Internet of medical things”) OR TITLE-ABS-KEY (“Artificial intelligence in internet of medical things”) OR TITLE-ABS-KEY (“Deep learning in internet of medical things”) OR TITLE-ABS-KEY (“Machine learning in internet of medical things”) OR TITLE-ABS-KEY (“Feature selection in internet of medical things”) OR TITLE-ABS-KEY (“Convolutional neural network in internet of medical things”) OR TITLE-ABS-KEY (“deep neural network in internet of medical things”) OR TITLE-ABS-KEY (“recurrent neural network in internet of medical things”) OR TITLE-ABS-KEY (“robots in internet of medical things”) OR TITLE-ABS-KEY (“swarm intelligence in internet of medical things”) OR TITLE-ABS-KEY (“Big data in internet of medical things”) OR TITLE-ABS-KEY (“internet of medical things and neural networks”).

The bibliometric data required for the analysis were ascertained based on the chosen bibliometric analysis techniques. The study selected the productivity of the research field constituents. As a result, the data on the number of publications originating from different countries were collected in the search results returned. The AMSTAR guidelines suggested that at least two databases should be used for data collection (Shea et al., 2007). However, a recent guideline proposed by Donthu et al. (2021) suggested using only one database to avoid the extra effort of harmonizing the different formats of multiple databases, which has the likelihood of introducing human error in the harmonization process. In the study by Bettera et al. (2022), only one database was used for the bibliometric study. Therefore, the study selected only the Scopus database to collect the required data for the bibliometric analysis. The data collected from the Scopus database were cleaned by removing erroneous data, unnecessary entries, and duplicates.

Scopus is a widespread database with comprehensive scientific data and literature (Boyle and Sherman, 2006). Hence, the datasets used in this study were obtained from the Scopus database. We gathered bibliographical information, citations, abstracts, keywords, and funding details that were relevant to IoMT research from 2016 to 2023. We used keywords as described in Section 3.4 to retrieve specific literature. Journal articles, conference papers, reviews, book chapters, notes, editorials, and short surveys were retrieved from the Scopus database. The datasets used to show the analytical method were chosen from 1,347 bibliographic entries published between 2016 and 2023. The factors examined were author, title, abstract, document type, publication year, language, nation, institution, citations, and worldwide collaboration.

### 3.5 Running the bibliometric

This is the last stage of the bibliometric methodology, where the study runs bibliometric analysis. The bibliometric software used for the study is VOSviewer because of its wide acceptability in the research community. The VOSviewer was run to generate both visual structure and summaries. The visual network was separated into clusters, and bibliometric summaries were generated. The findings collected and compiled from running the VOSviewer were presented and discussed in the next section.

## 4 Results and discussion

The results of the data analysis are presented in this section. The bibliometric analysis results involved both performance analysis and scientific mapping, as has already been discussed. The next sub-section discusses the results.

### 4.1 Distribution of publication types

**Figure 3:**
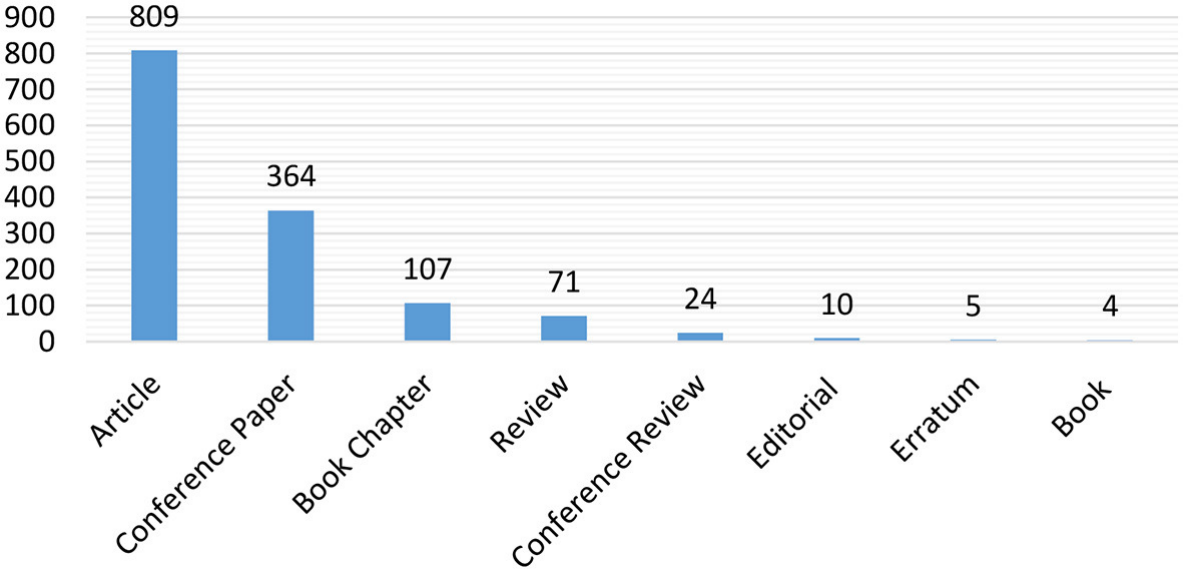
The distribution of the document types publishing the research on the applications of AI in the IoMT.

Figure 3 presents the distribution of document types in which research on AI in the IoMT was published. The graph shows that the largest contributions of the latest research results on AI in the IoMT were published in journals, indicated by the longest bars. The next most common publishing forum after the journal is conferences, while books recorded the lowest number of publications in this domain. This is likely because publishing books takes more time, so researchers prefer journals and conferences to disseminate their findings quickly. As this research area is emerging with many dynamics, delaying the publication of research results may be a disaster for researchers, as other research groups may publish similar results. The combined total of conference and journal articles indicates that researchers heavily rely on these platforms to publish the latest research outcomes on solving IoMT problems using AI. Therefore, researchers willing to contribute to this field should largely rely on journals and conferences to access complete, accurate, and reliable literature. This approach can greatly help researchers make new discoveries and advance the IoMT field.

### 4.2 Productivity by countries

**Figure 4:**
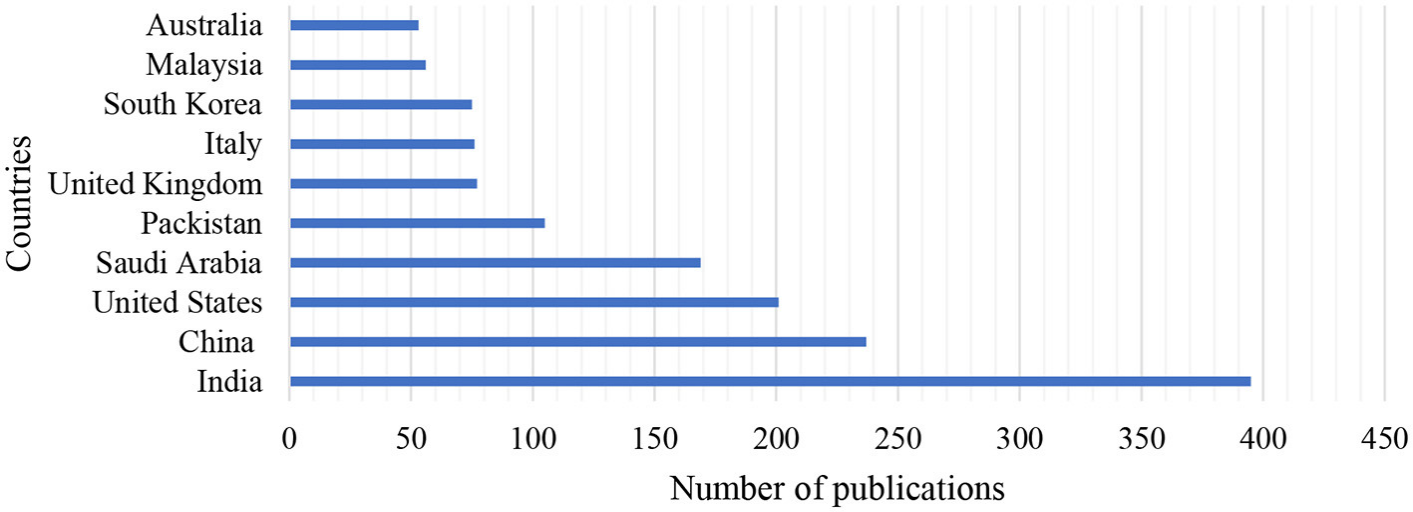
The ranking of the top 10 countries with the highest number of contributions.

Figure 4 depicts the distribution of publications originating from different countries during the period of 7 years. It depicts the top 10 countries with the highest number of articles produced on the research conducted on AI in the IoMT. It is clearly indicated that researchers from India contributed more articles in this domain than other countries on the list. China and the United States of America occupied the number 2 and 3 spots, respectively, for their contributions to the development of the research area. The lowest contributor in the top 10 countries is Australia, which has the shortest bar and indicates the lowest contributions. It can be observed that no single country from Africa is ranked in the top 10 list. Further observations indicate that countries from the Asia continent dominated the list, and the top two countries were all from Asia. Evidence revealed that there is global convergence in the development of this research area because different countries are engaged in the research, making it international and multi-dimensional.

### 4.3 Corresponding author countries

**Figure 5:**
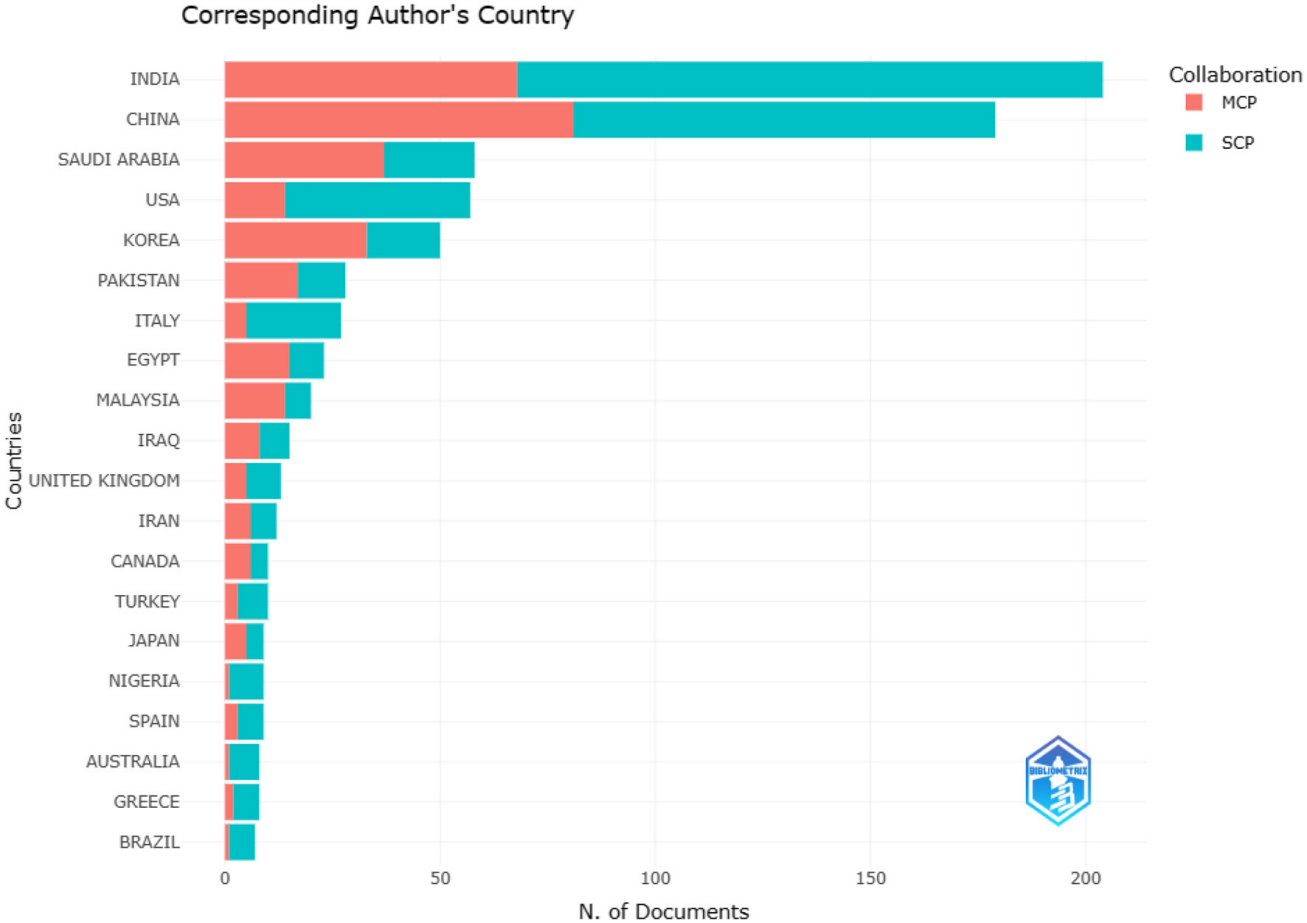
Top number of documents by countries publishing in the field of AI in the IoMT.

The corresponding author is typically critical when submitting a research manuscript for publication in a reputable journal. It is assumed that the corresponding author is the most senior researcher who supervised the research procedure, ensuring that all protocols and ethics in research were followed, and is responsible for the correspondence with the journal. In this study, the top 20 countries of corresponding authors have been depicted in Figure 5, indicating the number of corresponding authors from each country. It was found that India tops the list with the highest number of corresponding authors, followed by China and Saudi Arabia in the second and third positions, respectively. Countries from Asia dominated the top 20 countries. From the perspective of Africa, only Nigeria and Egypt make the list. The analysis indicates that authors from India, China, and Saudi Arabia were actively involved in the research and publication of the findings. Figure 5 illustrates the association of the documents with the countries. The documents in the dataset are each associated with only one country. This makes it possible to calculate metrics for publications that come from only one country (SCP) and those from multiple countries (MCP). By using the MCP ratio, we can gain insight into how much countries collaborate.

### 4.4 Research fields ranked by the percentage of publications

**Figure 6:**
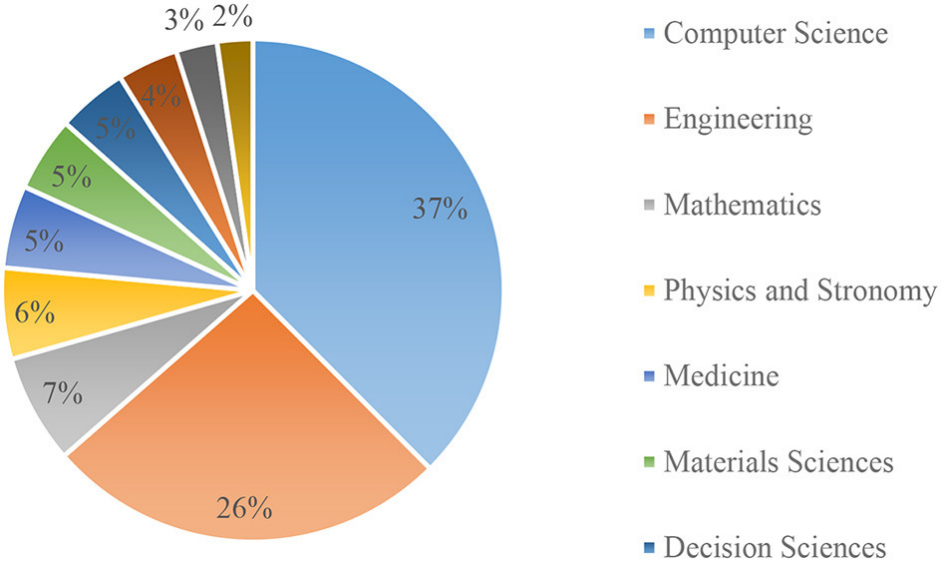
Contributions to the IoMT based on disciplines.

The AI in IoMT is multidisciplined. As shown in Figure 6, the distribution of contributions from different disciplines indicated that most of the contributions come from computer science. This is not surprising, as the origins of IoMT and AI are both research fields in computer science. The IoMT is at the intersection of computer science and healthcare. The analysis clearly shows that research in the IoMT can be conducted from a different perspective than computer science and medicine. This multidisciplinary nature of the research area can foster collaboration with researchers from different backgrounds.

### 4.5 Average citations per year

**Figure 7:**
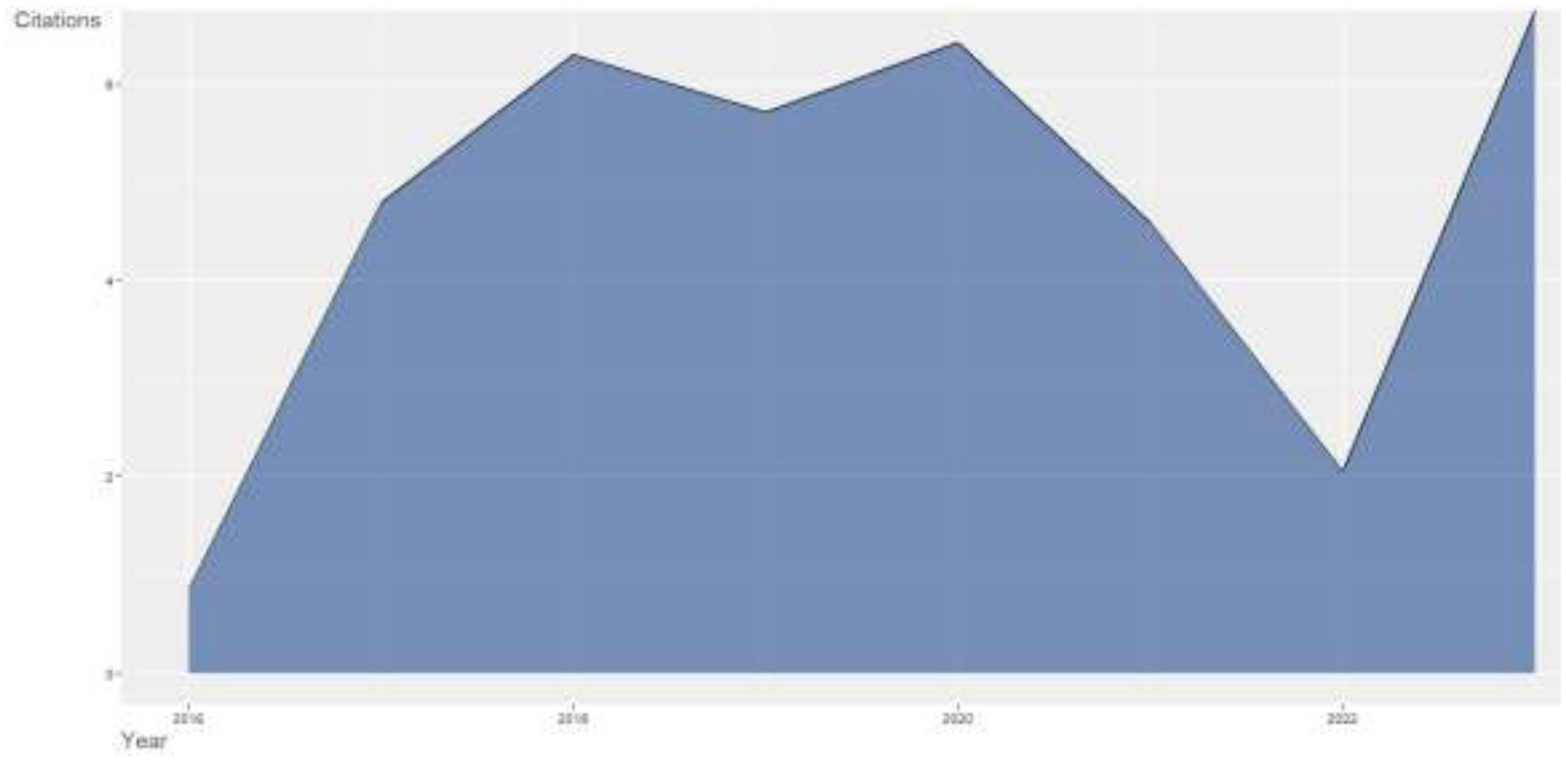
Average citations per year.

The impact of research is measured by the number of citations accumulated by the publication over a period of time. Figure 7 depicts the average citations accumulated per year for the research publications in AI in the IoMT over the course of 7 years from 2016 to 2022. The graph indicates that the number of citations has continued to grow since 2016. The year 2016 had the lowest number of citations but kept on growing over time. The citations for 2023 cannot be concluded because the year is still early. Table 3 presents the mean total citation per article (MeanTCperArt), year total citation per year (MeanTCperYear), and citable years (CitableYears).

**Table 3:** Total citations per article, year, and years.

**Year**	**N**	**MeanTCperArt**	**MeanTCperyear**	**Citableyears**
2016	4	6	0.857142857	7
2017	17	28.76470588	4.794117647	6
2018	54	31.48148148	6.296296296	5
2019	121	22.84297521	5.710743802	4
2020	214	19.27102804	6.423676012	3
2021	393	9.155216285	4.577608142	2
2022	520	2.053846154	2.053846154	1

### 4.6 Most relevant sources of literature

Many journals have published studies in this domain, making it impractical to list all the journals/venues publishing studies. Therefore, the top 20 sources of relevant literature were ranked according to productivity. Table 4 presents these top 20 sources. It is shown that the IEEE Access journal tops the ranking of the literature sources, followed by IEEE Internet of Things and Sensors in the second and third positions, respectively. The journal with the lowest number of publications in this field is IEEE Transactions on Consumer Electronics, which is not surprising given that its focus is not mainly on AI in the IoMT. It can be observed that most forums publishing studies on AI in IoMT are computer science forums, followed by medical journals within the scope of informatics. The IEEE forum dominated the ranking of sources publishing research on AI in IoMT. This ranking can clearly reveal to researchers and industry practitioners where to retrieve credible, accurate, complete, and updated information about the research conducted in the field of IoMT.

**Table 4:** The sources of literature for publishing studies on AI in IoMT.

**Sources**	**Articles**
IEEE Access	83
IEEE Internet of Things Journal	64
Sensors	36
IEEE journal of biomedical and health informatics	32
Future-generation computer systems	24
Electronics (Switzerland)	23
IEEE transactions on industrial informatics	21
Computer communications	18
Internet of Things	18
Computers and electrical engineering	16
Lecture notes in networks and systems	16
Communications in computer and information science	15
Computer materials and continua	15
Computational intelligence and neuroscience	14
Sensors (Switzerland)	14
Studies in computational intelligence	13
Lecture notes in electrical engineering	12
IEEE transactions on computational social systems	11
Lecture notes in computer science (including subseries lecture notes in artificial intelligence and lecture notes in bioinformatics)	11
IEEE transactions on consumer electronics	10

**Figure 8:**
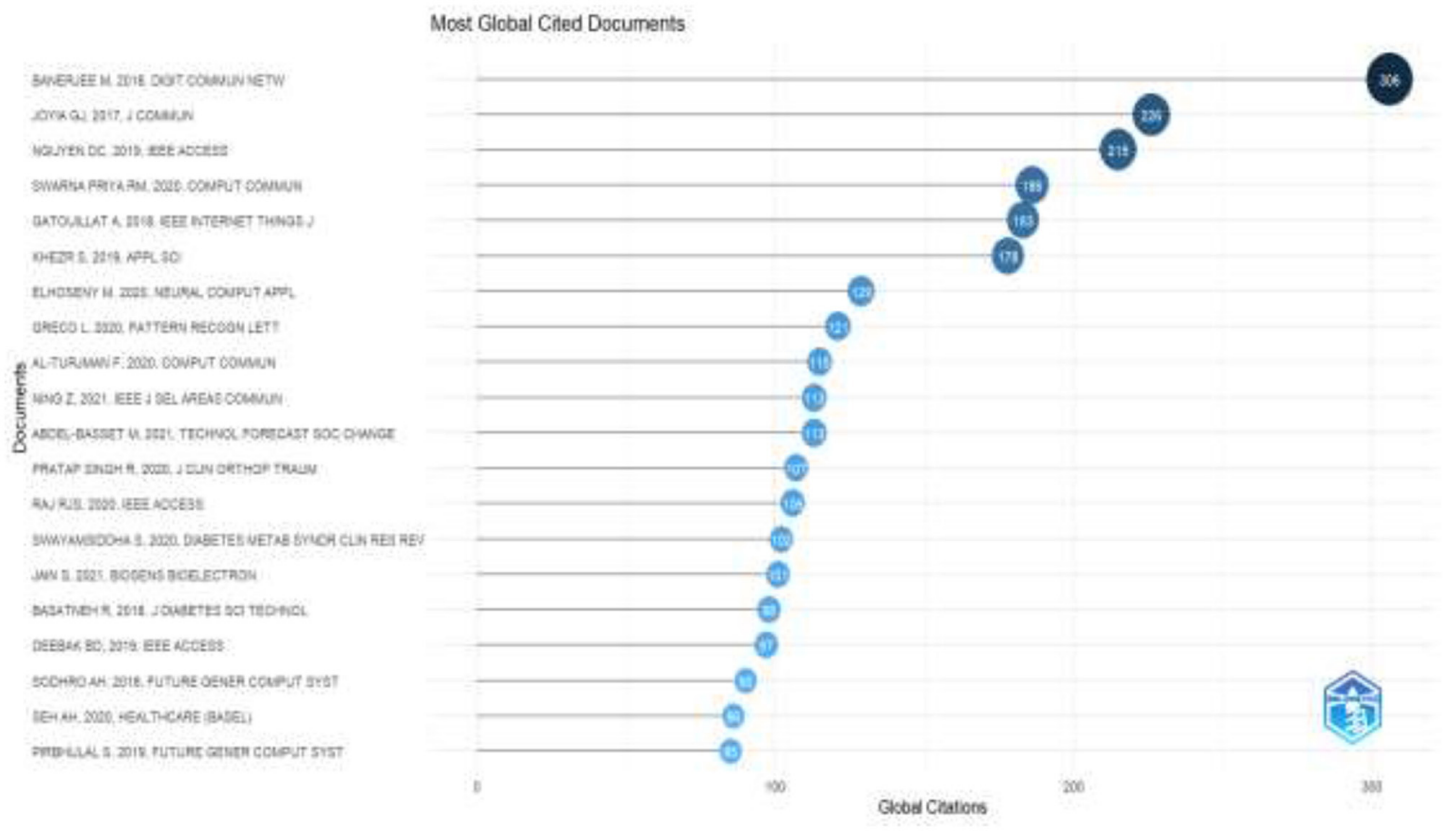
Most globally citable documents.

Figure 8 depicts the most citable documents globally in the field of AI for IoMT. It can be observed that the top citation document is “*Banerjee M. 2018 Digit commun Netw*.” However, the IEEE forum dominated the ranking of the top citable documents.

### 4.7 Authors' productivity

Table 5 presents an example of the productivity of the top 10 authors publishing in the research area, along with the corresponding articles' fractionalization. It can be observed from the top list that Mohanty SP has the highest productivity among the top contributing authors. On the other hand, Chen J., Kumar A., and Lakhan A. are among the least productive of the top authors. Potential authors willing to collaborate in the area of AI in IoMT can easily identify the authors to contact for the purpose of collaboration, as the identities of the top authors are revealed in the study.

**Table 5:** Top 20 productive authors.

**Authors**	**Articles**	**Articles fractionalized**
Mohanty SP	26	6.85
Kougianos E	21	5.38
Guo J	20	4.47
Khan MA	15	2.59
Pirbhulal S	13	2.55
Wang W	13	2.53
Wang X	13	2.32
Chen J	12	2.09
Kumar A	12	3.12
Lakhan A	12	2.08

### 4.8 Authors' institutional affiliation

**Figure 9:**
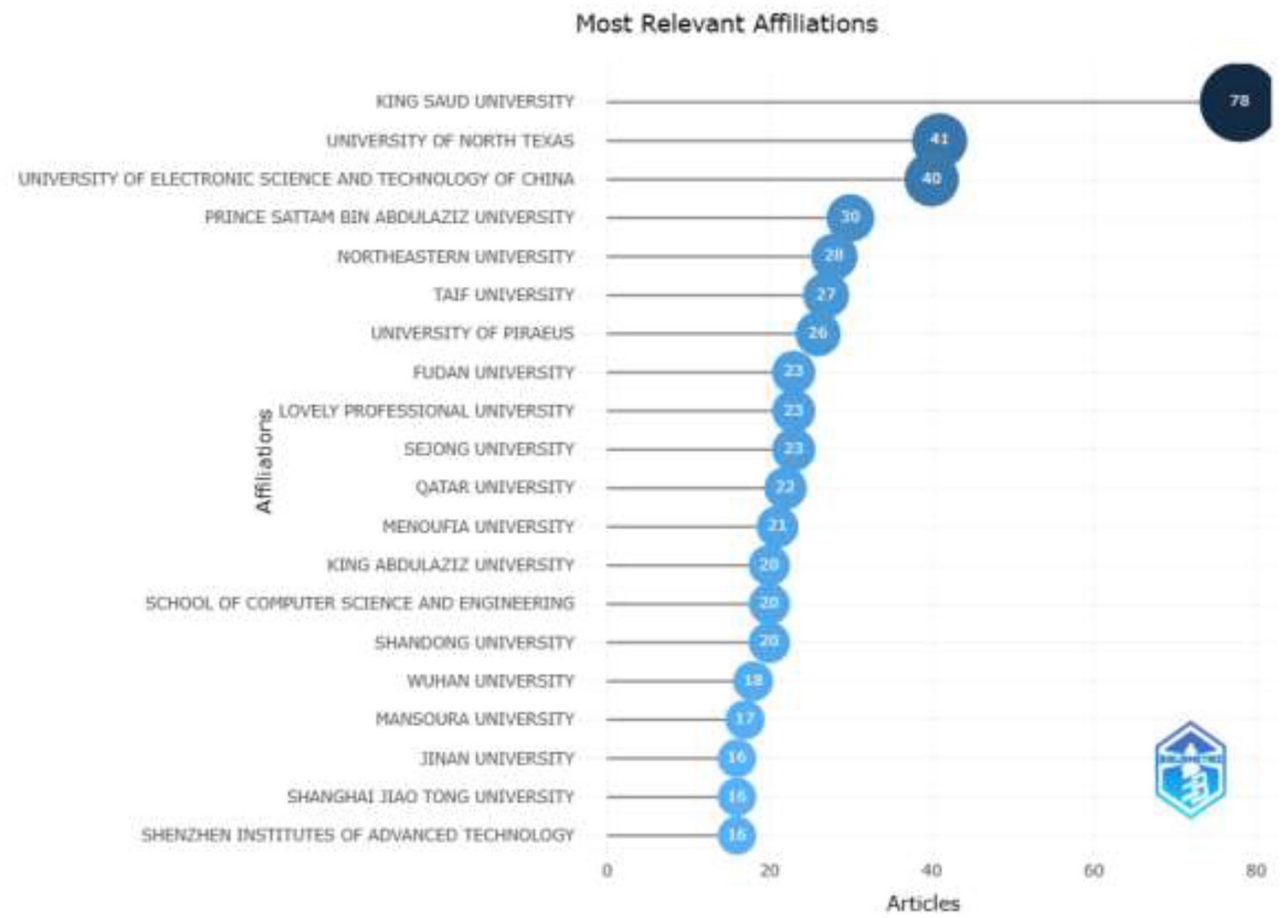
Top 20 authors' institutional affiliations.

Figure 9 shows the top 20 authors' institutional affiliations in the world, indicating where authors publishing research in the area of AI in IoMT are based. The longest bar represents the institution with the highest proportion of affiliated authors. King Saud University in Saudi Arabia is the top institution publishing in the field of AI in IoMT, followed by the University of North Texas in the United States and the University of Electronic Science and Technology, China, occupying the second and third spots, respectively. Further observations indicate that the top 20 institutions were dominated by institutions from China. At the continental level, most of the institutions are located in Asia, making it the hub of research in AI for IoMT. From the perspective of Africa, only institutions from Egypt make the list of the top 20 institutions.

### 4.9 Most frequent words

Table 6 presents the top 20 keywords mostly used by authors in the literature, along with the occurrences of these keywords across the analyzed documents. The analysis indicates that the following keywords are the most common: “Internet of Things,” appearing 453 times, making it the top keyword, followed by “healthcare” in the second position, then “Internet of medical things,” “diagnosis,” “deep learning,” “human,” “blockchain,” and “Internet, and others.

**Table 6:** Top 20 author-keyword occurrences.

**Words**	**Occurrences**	**Words**	**Occurrences**
Internet of Things	453	Machine learning	113
Health care	337	Humans	107
Internet of medical thing	292	Security	97
Diagnosis	209	Diseases	93
Deep learning	172	Data privacy	92
Human	138	Edge computing	89
Blockchain	123	Learning systems	87
Internet	120	Medical imaging	87
Network security	120	Cryptography	81
Artificial intelligence	118	Authentication	77

**Figure 10:**
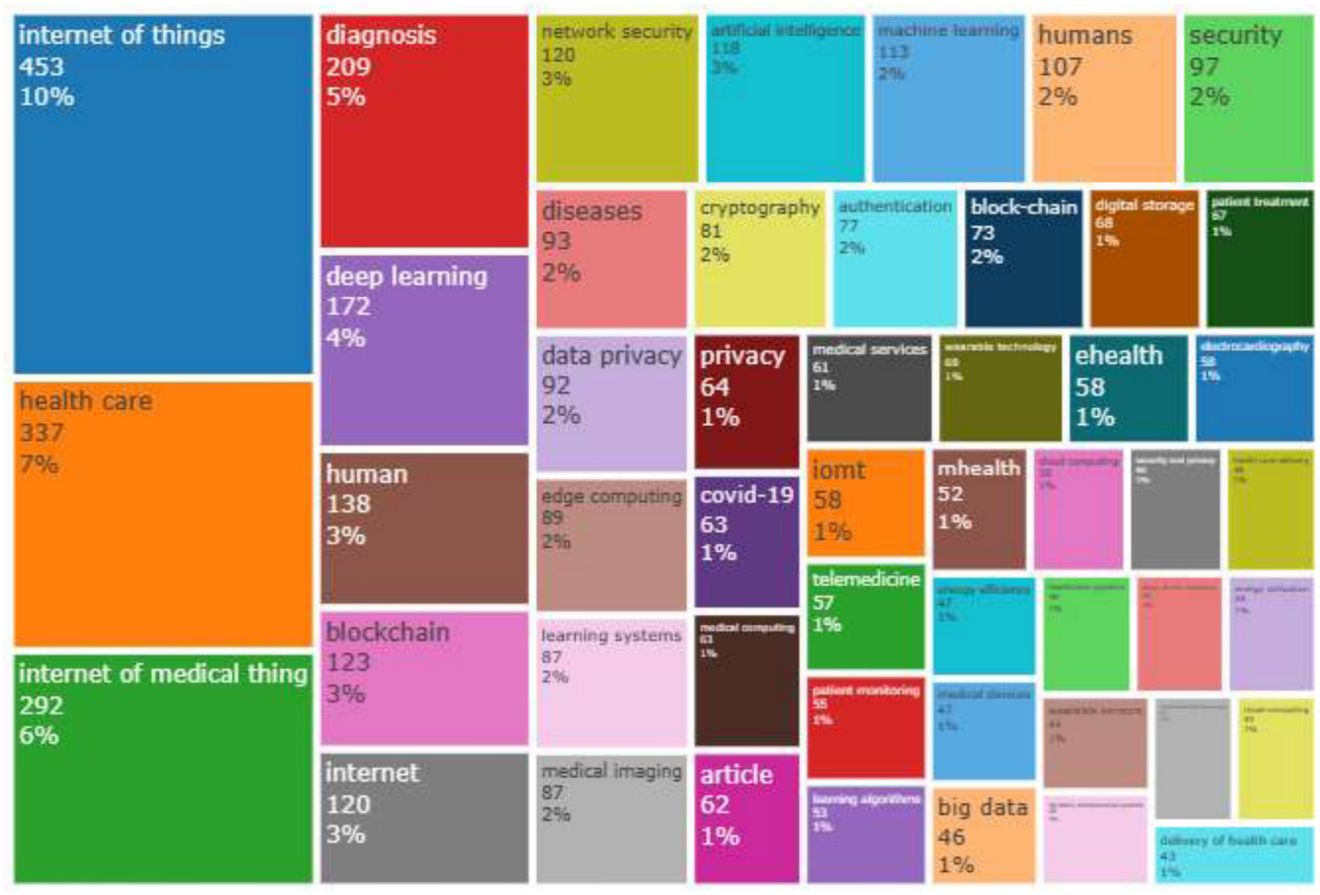
Treemap of articles based on the keywords.

Figure 10 presents the tree map of articles based on the keywords. The area of the rectangles indicates the frequency of the words. For example, the top left subject matter is “Internet of Things,” “healthcare,” “diagnosis,” and “deep learning.” In the top right, the topic focused more on techniques and technologies such as “AI,” “machine learning,” “blockchain,” and others. In addition, the bottom topics indicate topics such as “big data,” “Internet of Medical Things,” and more.

### 4.10 Trending topics

**Figure 11:**
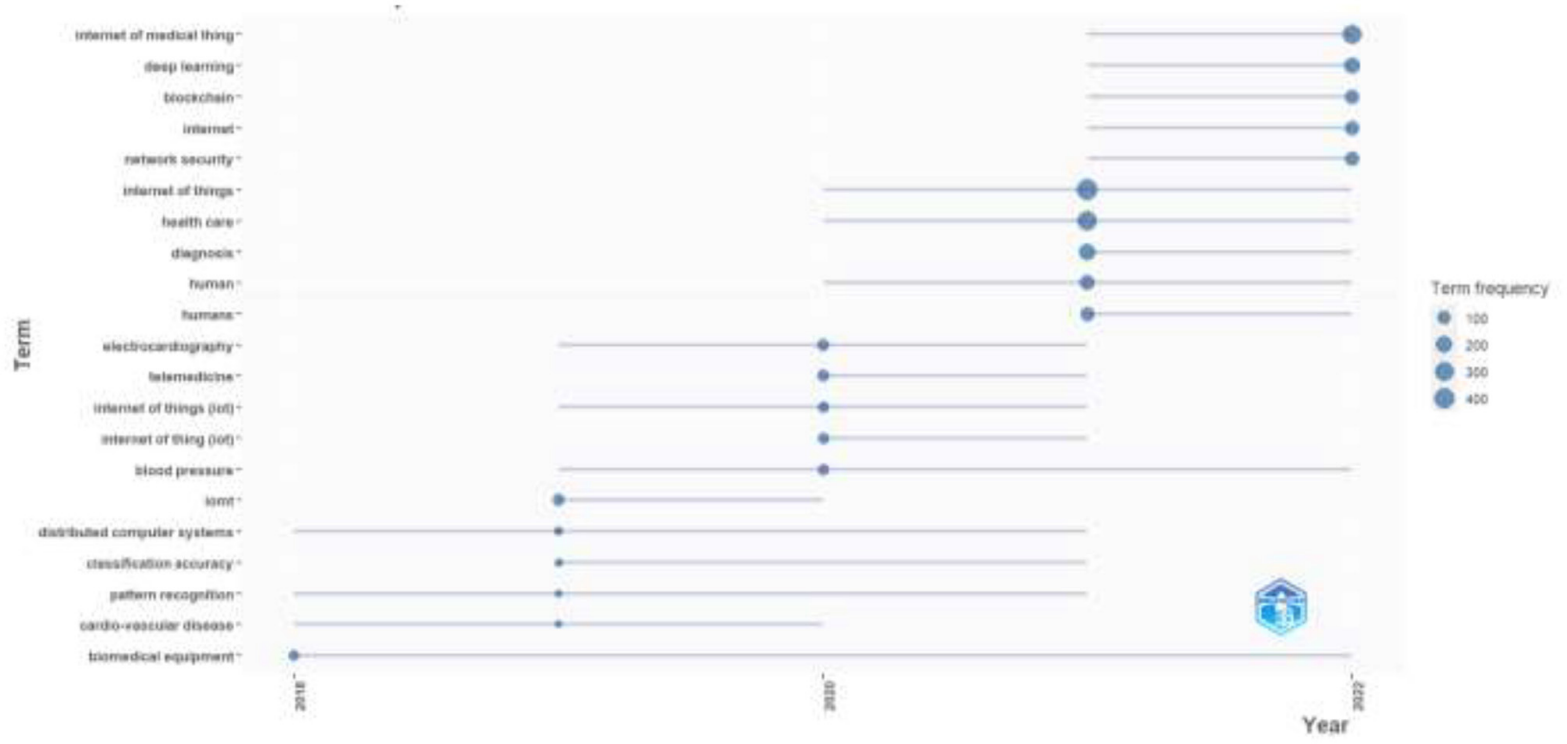
Topics trending in the research field.

**Figure 12:**
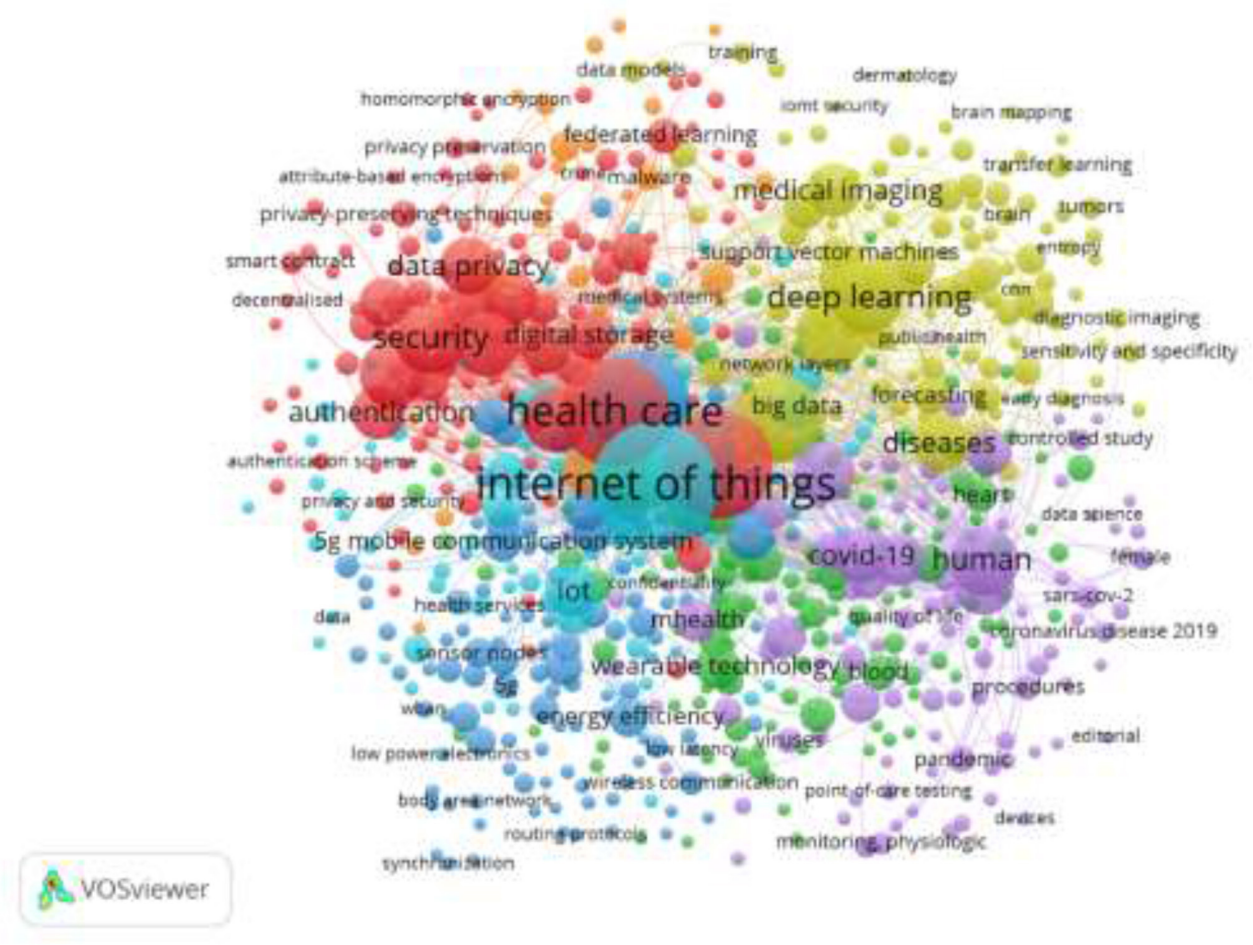
Co-word appearance.

The topics that are currently popular or discussed in AI in the IoMT research field were extracted and presented in Figure 12 to show the pattern of the trending topics. Figure 11 depicts the top 20 trending topics. It can be observed that the most trending topic is the IoMT, which signifies the popularity of the research area in the research community. The second spot is deep learning, a research field in AI. This is expected as the research scope combines AI and the IoMT.

### 4.11 Co-words (popular keywords)

Figure 12 depicts the co-word network structure visualization analysis, showing four major clusters in the network structural mapping. Each of the nodes in the network structure represents the occurrence of the keywords, where the popularity of the keywords is represented by the size of the node associated with the keyword. This means that the size of the node is directly proportional to the frequency of keyword occurrences. It is observed that each of the nodes is connected to another node, indicating the co-occurrences that exist between the keywords, meaning those words that occurred together in the literature repeatedly. The thicker the link between keywords, the more frequent the co-occurrences. As observed earlier, four main clusters exist in the network visualization: in the first cluster, the combination of “security and privacy” shows more frequent co-occurrences; in the second cluster, “healthcare and IoT.” The third cluster is “deep learning and medical image,” and the fourth cluster is “COVID-19 and human.” These were the prominent combinations of the keywords that appeared frequently in the AI in IoTM literature.

### 4.12 Author keywords

**Figure 13:**
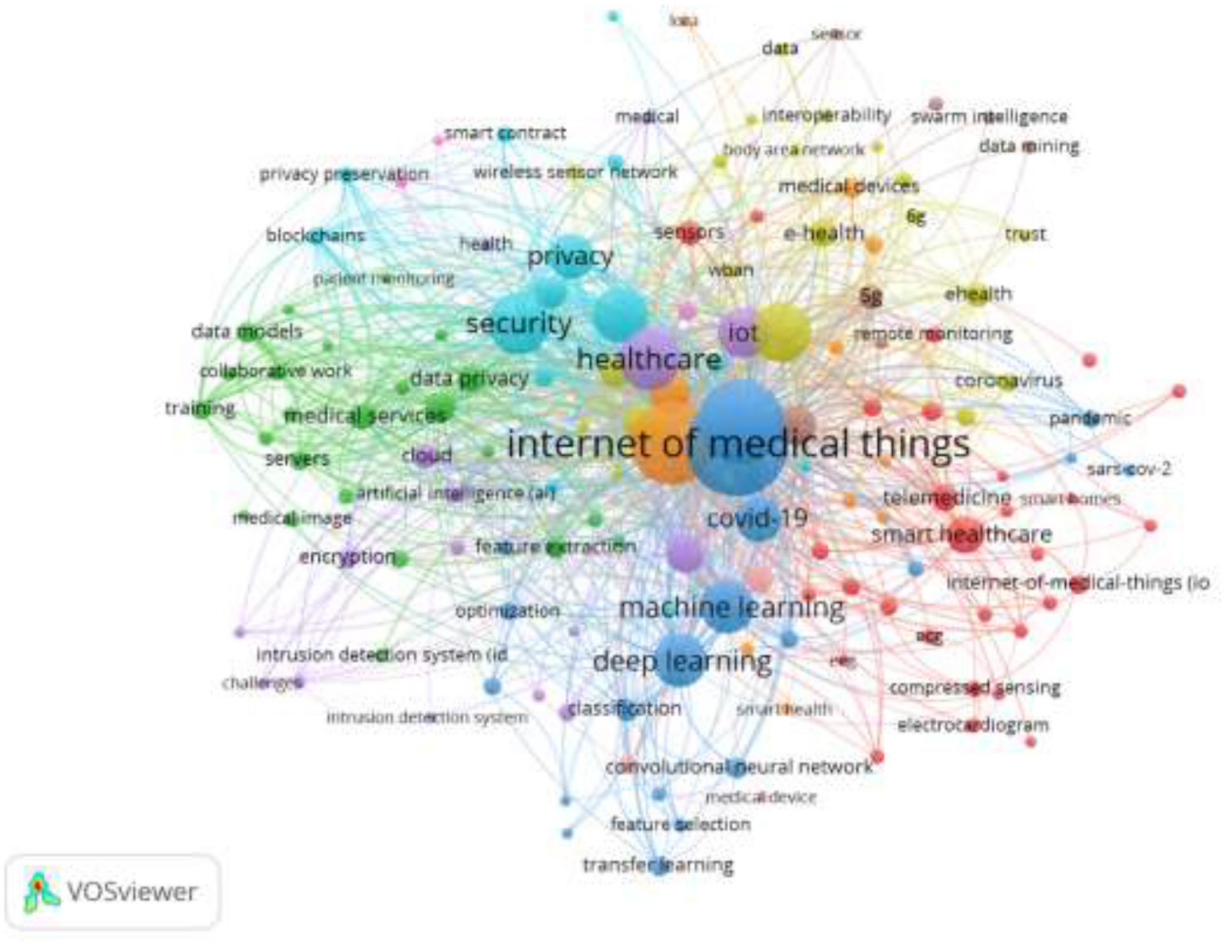
Author keyword visual network structure.

The author keywords typically show the predominant keywords frequently used by the authors publishing in the area of AI in IoMT. Figure 13 is a visual representation of the popular keywords among the authors. The thickness of the node indicates the intensity of the author's keywords. It is obvious that the keywords IoMT and healthcare, as well as security and privacy, dominated the author's keywords. The author's keyword network visual structure created six major clusters. The first cluster was dominated by the keywords medical device and e-health. The second cluster is data privacy and medical services, the third cluster is challenges and encryption, the fourth cluster is machine learning and deep learning, the fifth cluster is smart healthcare and telemedicine, and the sixth cluster is security and privacy. The author's keywords, mostly found in the network structure, can be classified roughly into AI, health, security, and privacy. These keywords can provide direction to new researchers or researchers intending to start work in the area on the keywords to use to search for relevant literature.

### 4.13 Co-authorship analysis

**Figure 14:**
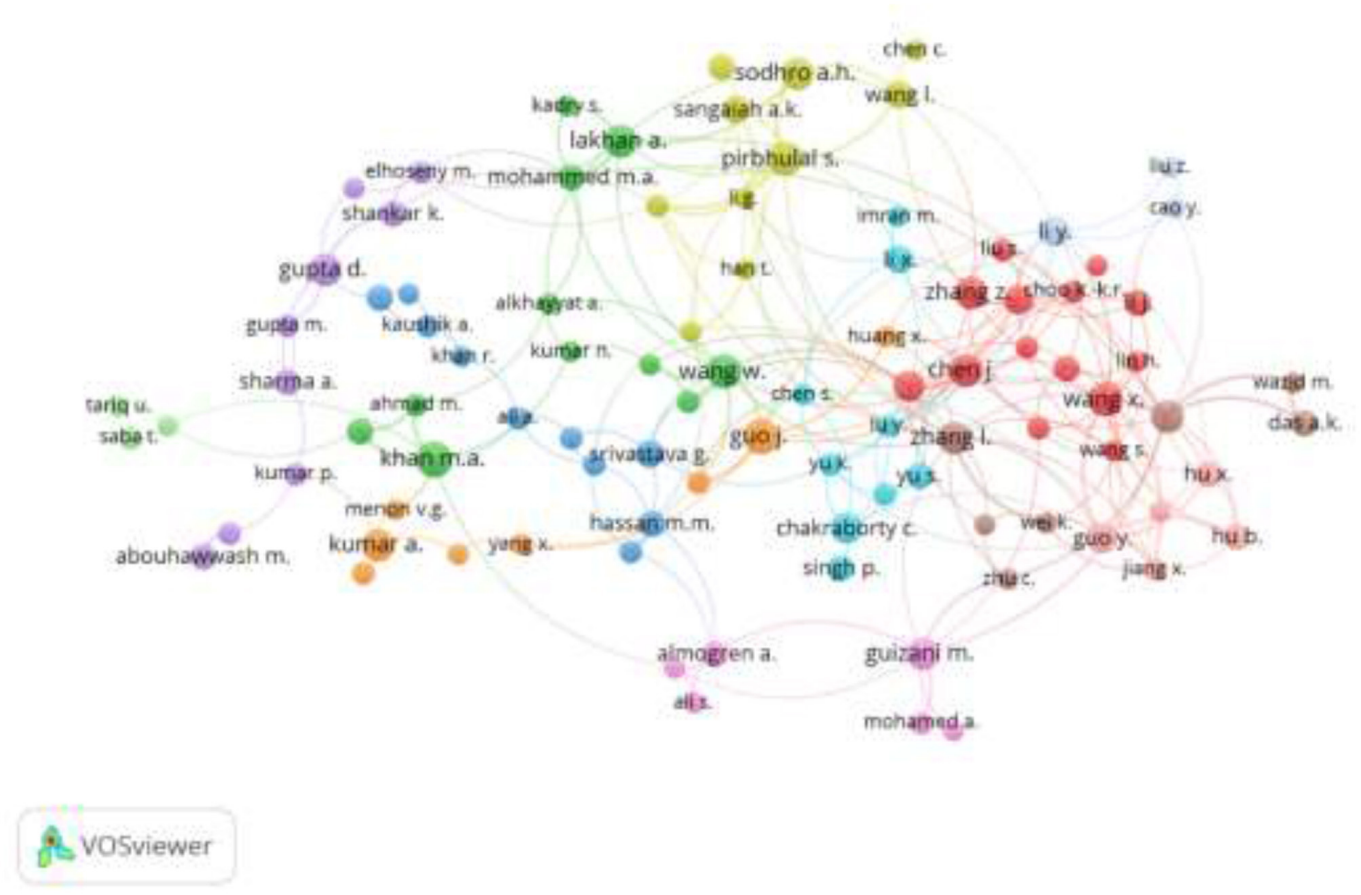
Bibliometric co-authorship coupling.

**Figure 15:**
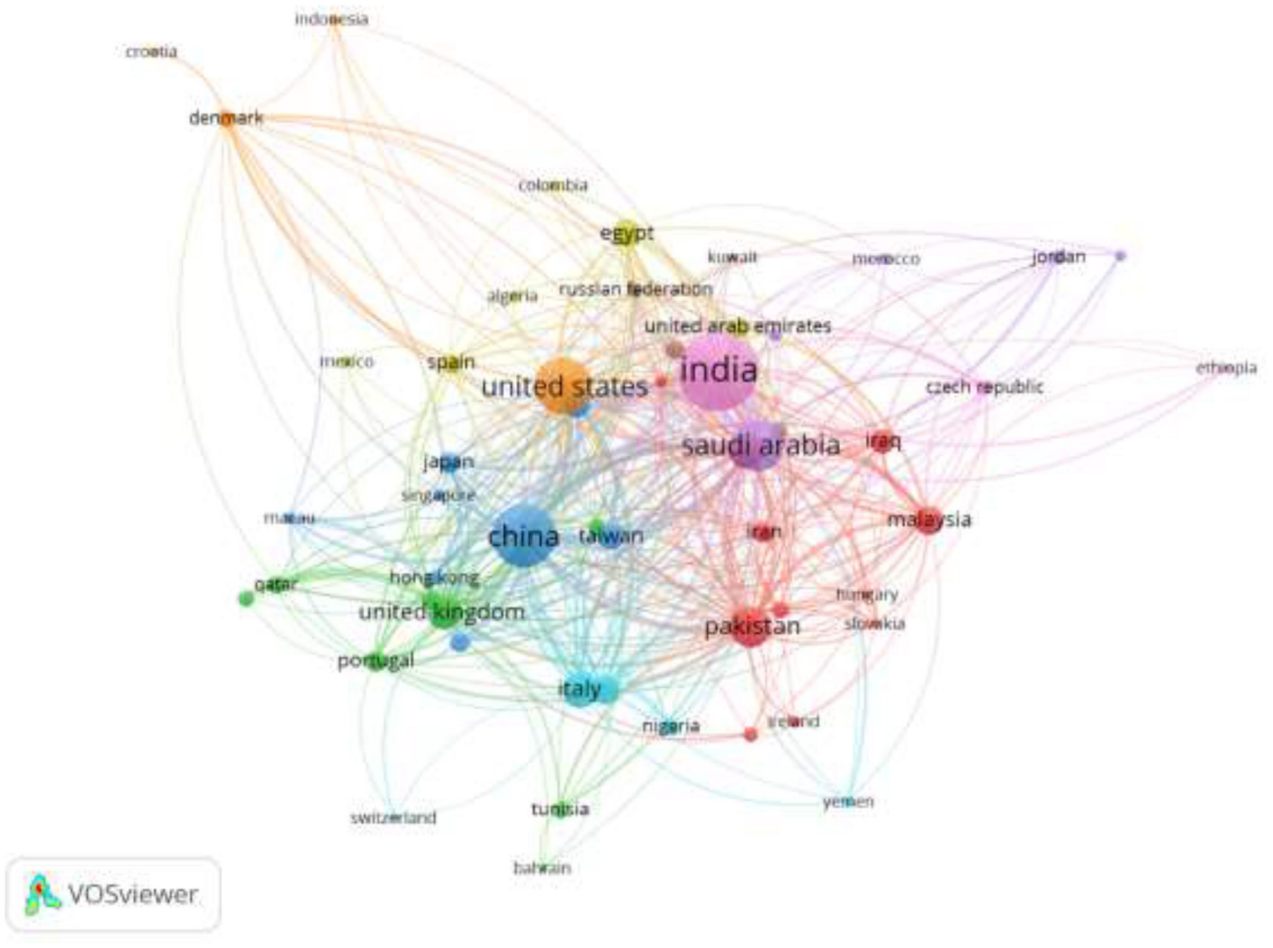
Bibliometric mapping Coupling the author's countries.

Figure 14 presents the relationship among the authors publishing in the area of AI in IoMT. The co-authorship network structure shown in Figure 15 depicts the co-authorship among the researchers publishing in the area of AI for IoMT. Each node in the figure represents an author, and the link connecting the nodes represents the co-authorship between the authors. The thick lines indicate the intensity of the co-authorships between the authors. The co-authorship network structure forms three major clusters. Cluster 1 shows that Pirbhulala S., Sangaiah A.K., and Sodhro A.H. had the biggest node in the cluster, signifying as the main authors, whereas in Cluster 2, Zhang Z., Chen J., and Wang X. were the main authors. In cluster 3, Guo and Hu were the main authors.

### 4.14 Collaborating countries

The authors' location at their affiliate institution determined the collaborating countries. Figure 15 depicts the collaborations among the affiliated countries for the authors publishing in this research field. Each node represents a country, and the links between each of the nodes indicate collaborations among those countries. The bigger the node, the higher the number of collaborations with the country; likewise, the thickness of the link connecting the countries indicates the intensity of the collaborations. The five major countries across the five different clusters are the United States of America, India, China, Saudi Arabia, and Pakistan, indicating a high number of authors' affiliated institutions in those countries. Among the countries, India has the biggest node, indicating that India serves as the hub for collaborating countries publishing in the field of AI in IoMT over the last 7 years, covering the study period.

### 4.15 Discussion and implication

A bibliometric analysis was conducted for the research area at the intersection of AI and IoMT, where AI tools were applied to improve the effectiveness and efficiency of the IoMT. The performance analysis and scientific mapping in this field analyze recent trends in the applications of AI to solve problems in IoMT. The study provides guidance and motivation for future research, along with mapping the network structure in the AI for IoMT research area. New and novice researchers willing to start research in this domain and expert researchers seeking new discoveries in the area of AI in IoMT can highly rely on journals and conference proceedings to conduct studies. This is because researchers working in this domain rely heavily on journals and conference proceedings to publish their latest research results. The study has identified trending topics within the research community, which can inspire individuals to explore this research area with the potential for groundbreaking discoveries that benefit both the research community and society at large.

Researchers working in this research field can use the study to easily identify trending topics and conduct research in similar areas. The study has clearly revealed that the use of AI for solving problems in IoMT may likely continue growing and remain relevant, especially with the renewed and unprecedented interest in AI that has been generated by the media and the general public. Policymakers in different countries can use the study to gauge their research productivity and impact in the area of AI applications for IoMT. This can help them determine the next line of action for their country's research in AI for IoMT. The study has proven that this research area is multidisciplinary. Researchers from disciplines other than computer science and healthcare willing to start research in this area can use the study to assess the suitability of their discipline or research area's potential to contribute to the development of IoMT. Countries interested in emerging medical services delivery mediums can find the study useful for directing funding toward high-impact research in the field and can easily identify potential collaborating institutions, countries, and authors.

Most of the authors' collaborating institutions were found to be domiciled in India, making India the hub of collaboration for research regarding AI in IoMT. However, King Saud University in Saudi Arabia is the top institution, with the highest number of authors publishing in the research area. The best possible reason some countries and institutions are more inclined to work in this domain is that the countries and institutions have likely established research expertise in technology to enhance the healthcare domain, access to funding and resources, favorable regulatory environments, participation in collaborative networks, and unique healthcare challenges requiring advanced technology. Publishing houses can use this study to gauge the performance of their journals and conference proceedings in publishing research on applying AI to solve problems in the IoMT domain.

Research organizations, funding bodies, medical organizations, governments across the world, and ranking bodies can use this study to identify relevant information in the area of bibliometric analysis for AI in IoMT. This study undoubtedly indicates that the issues of security and privacy are serious concerns, hindering the massive adoption of the IoMT. The literature review presented in Section 2 and the bibliometric analysis of the subject matter visibly attest to these concerns. We believe that understanding the applications of AI in IoMT through the performance analysis and science mapping conducted in this article can facilitate the discovery of new knowledge in the research field. The areas in AI that have not yet significantly impacted IoMT include generative AI, brain-machine interfaces, artificial general intelligence, and explainable AI (XAI).

## 5 Futuristic direction from a different perspective

The bibliometric analysis shows that success has been recorded in the use of AI techniques to solve issues in the IoMT. However, different aspects of AI remain underexploited or unexploited in solving IoMT problems. In this section, we presented future directions from the perspective of different types of AI rather than focusing solely on AI-specific improvements for the IoMT.

### 5.1 Explainable artificial intelligence

This section discusses the perspective on the integration of explainable artificial intelligence (XAI) into the IoMT and explains the motivation behind the need for XAI in this context.

It can be observed that, despite the growing acceptability of XAI in the research community, it has yet to make significant inroads into the IoMT. XAI has recently been developed to address the limitations of conventional AI, such as the lack of explanation, transparency, and interpretability. Although AI has achieved significant success in various application domains, the opaque processes used by AI models to reach certain decisions remain a challenge. To resolve this challenge, XAI incorporates abilities like context-specific learning, reasoning, and abstract thinking. One of the AI algorithms that has received serious backlash for its “black box” nature is artificial neural networks, especially their advanced variant with the new generation of neural networks, referred to as deep learning (DL). With the advent of XAI, it is now possible to extract relational explanations for DL, as is evident in the study by Townsend et al. (2019).

The XAI has begun gaining significant interest from academia and industry in different domains such as medical (Biffi et al., 2020), engineering design (Sasaki et al., 2021), the Choquet integral (Murray et al., 2020), and more. However, our focus in this study is the IoMT domain because of its importance and significance to healthcare delivery in society.

However, the integration of XAI into the IoMT is scarce in the literature, and researchers have hardly made any efforts to connect XAI and the IoMT. If the robot decides to perform surgery on a patient, the patient would like to know why the decision was made. Or if the robot orders amputation, will the patient be interested in recognizing exactly the explanation behind the decision? If an intelligent algorithm performs diagnosis and prediction, the user would like to know the explanation of the salient feature used by the algorithm to reach its decision. The application of XAI to IoMT to process data should be the future focus of researchers working in the domain. Introducing XAI into IoMT could increase patient confidence and the acceptability of the decisions made by IoMT. Incorporating XAI into IoMT can provide interpretation, explanation, transparency, and accountability for the AI models used in processing the data collected from IoMT on how certain decisions are made.

### 5.2 Artificial general intelligence

Artificial intelligence, particularly in the IoMT, can use multiple machine learning algorithms for various applications, including disease prediction, remote health monitoring, human behavior prediction, automatic insulin injection, intelligent medicine boxes, mental health monitoring, sleeping monitoring, seizure detection, fall detection, stress, and anxiety monitoring (Ashfaq et al., 2022). In contrast, artificial general intelligence (AGI) can perform all these tasks within a single framework. Developments in the area of AI led to the creation of AGI-based systems with the capability to perform any task typically associated with intelligent agents, especially human beings. AGI is expected to improve decision-making processes by handling and analyzing large-scale data more accurately and objectively than human decisions. AGI is believed to have the capacity to provide personalized services tailored to individual differences, preferences, needs, and feelings (IEEE Computer Society, 2023). AGI remains unexploited in the area of IoMT, as shown in previous studies. Some of the major issues hindering the widespread adoption of IoMT, such as security, privacy, and accuracy, could potentially be addressed by AGI. We suggest researchers explore this area vigorously in the future.

### 5.3 Generative artificial intelligence

Generative artificial intelligence (GAI) is an emerging research area within AI that involves training machine learning models to create content such as text, video, image, and voice instead of solving conventional machine learning problems such as prediction, classification, clustering, and association rules. ChatGPT is a typical example of an application built on GAI that is capable of generating content to enhance human productivity. Generative adversarial networks (GANs) are one of the core algorithms for content generation.

In the coming years, GAI is expected to gain unprecedented attention for its potential to improve effectiveness and efficiency and open ways for new services. However, it also raises concerns about issues like ethics and societal impact. GAI can open new application areas, increase machine autonomy, and enhance human creativity (IEEE Computer Society, 2023). GAI has the potential to introduce new solutions for the IoMT. Given that the IoMT generates vast amounts of data in different formats, GAI could be explored for drug discovery, pathogen sequencing, protein structure analysis, and vaccine development, potentially reducing the time required for their design and development. Ezugwu et al. (2021b) stated that machine learning is a promising area to explore in pharmaceuticals. We strongly suggest researchers explore GAI in IoMT in the future.

## 6 Conclusions

In this study, a bibliometric analysis comprising both performance analysis and scientific mapping was conducted for AI in IoMT. The performance analysis included institutions, countries, productivity, impact, and documents. Additionally, the network structure mapping covered co-keyword appearance, authorship analysis, countries, and author keywords. The past, present, and future directions for research in IoMT were outlined and discussed. The study found that security and privacy issues are serious concerns hindering the large-scale adoption of the IoMT. Future research directions from different points of view were highlighted.

## Author contributions

HC: Conceptualization, Data curation, Formal analysis, Methodology, Writing – original draft. IH: Conceptualization, Data curation, Formal analysis, Methodology, Writing – original draft. MM: Conceptualization, Data curation, Funding acquisition, Investigation, Writing – original draft, Writing – review & editing.
